# Effect of free-base and protonated nicotine on nicotine yield from electronic cigarettes with varying power and liquid vehicle

**DOI:** 10.1038/s41598-020-73385-6

**Published:** 2020-10-01

**Authors:** Soha Talih, Rola Salman, Rachel El-Hage, Nareg Karaoghlanian, Ahmad El-Hellani, Najat Saliba, Alan Shihadeh

**Affiliations:** 1 Mechanical Engineering Department, Maroun Semaan Faculty of Engineering and Architecture, American University of Beirut, Bliss Street, PO. Box 11-0236, Beirut, Lebanon; 2 Chemistry Department, Faculty of Arts and Sciences, American University of Beirut, Bliss Street, PO. Box 11-0236, Beirut, Lebanon; 3 Center for the Study of Tobacco Products, Virginia Commonwealth University, 821 West Franklin Street, Richmond, VA 23284 USA

**Keywords:** Risk factors, Health policy

## Abstract

Nicotine in electronic cigarette (ECIG) liquids can exist in a free-base or protonated (or “salt”) form. Protonated nicotine is less aversive upon inhalation than free-base nicotine, and many ECIG manufacturers have begun marketing protonated nicotine products, often with high nicotine concentrations. Regulations intended to control ECIG nicotine delivery limit nicotine concentration but do not consider nicotine form. In this study, we systematically examined the effect of nicotine form on nicotine yield for varying powers and liquid vehicles. A Kanger Subox Mini-C tank ECIG (0.5 Ω) was used to generate aerosols at varying powers (5–45 W) from liquid solutions that contained either free-base or protonated nicotine at 15 mg/g concentration, with a liquid vehicle consisting of either propylene glycol (PG) or vegetable glycerin (VG), resulting in four different solutions (free-base/PG, free-base/VG, protonated/PG, and protonated/VG). Nicotine yield was quantified using gas chromatography-mass spectrometry. Nicotine yields were not influenced by nicotine form under any condition investigated. At each power level, PG-based liquids resulted in approximately double the nicotine yield of VG-based liquids. Nicotine concentrations in the aerosols matched those of the parent liquids for both the PG and VG conditions. Increasing power led to greater nicotine yield across all conditions. The amount of nicotine emitted by an ECIG is independent of whether the nicotine is free-base or protonated, however the liquid vehicle has a strong effect on yield. Regulations intended to limit nicotine emissions must consider not only nicotine concentration, but also liquid vehicle and device power.

## Introduction

Nicotine in tobacco products can be found in a free-base or protonated (“salt”) form, depending on the pH of the product. In internal tobacco industry documents, nicotine form has been long recognized as central to the sensory experience of tobacco use, particularly in what is known as “impact”^1^. In the 1960s, Philip Morris began manipulating the ratio of free-base to protonated nicotine in cigarette smoke, a factor that is described as key to the ascension of the Marlboro brand to the status of the world’s top-selling cigarette^1^. In 2014, PAX Labs, the original maker of the JUUL electronic cigarette (ECIG), obtained a patent for mixing free-base nicotine with an acid to convert it to the salt form^2^. This formulation reduces the aversiveness associated with inhaling the high free-base nicotine liquids^3,4^. Using nicotine salts, JUUL was able to employ nicotine concentrations as high as 50 mg/mL at a time when ECIG products available on the market averaged a nicotine concentration of 12 mg/mL, predominantly in the free-base form^5,6^. The transition to a high salt-based nicotine concentration liquid allowed the JUUL manufacturers to design a device that emits a high nicotine yield in a small puff volume.

Today, the use of nicotine salts is rapidly growing, and many ECIG manufacturers offer nicotine salt-containing ECIGs and refillable solutions^7^. These devices and liquids vary by liquid composition, i.e., propylene glycol to vegetable glycerin (PG/VG) ratio, the two most common ECIG liquid vehicles^5^, electrical features, and device design. While ECIG nicotine yield has been shown to increase with PG/VG ratio and power^8,9^, the influence of nicotine form on yield previously has not been examined directly; the data available to date indicate that for a given pH, nicotine yield is independent of the acid used in the liquid^10^, and that the protonated to free-base ratio found in the ECIG aerosol matches that of the liquid^11,12^. This knowledge gap is salient because to date EU and proposed US regulations aiming to limit nicotine delivery focus exclusively on nicotine concentration^13^, neglecting form, PG/VG ratio, and electrical power, among other factors.

In this study, we examined the effects of free-base vs. protonated nicotine forms on nicotine yield and the amount of liquid aerosolized while varying electrical power and liquid vehicle.

## Results

**Figure 1:**
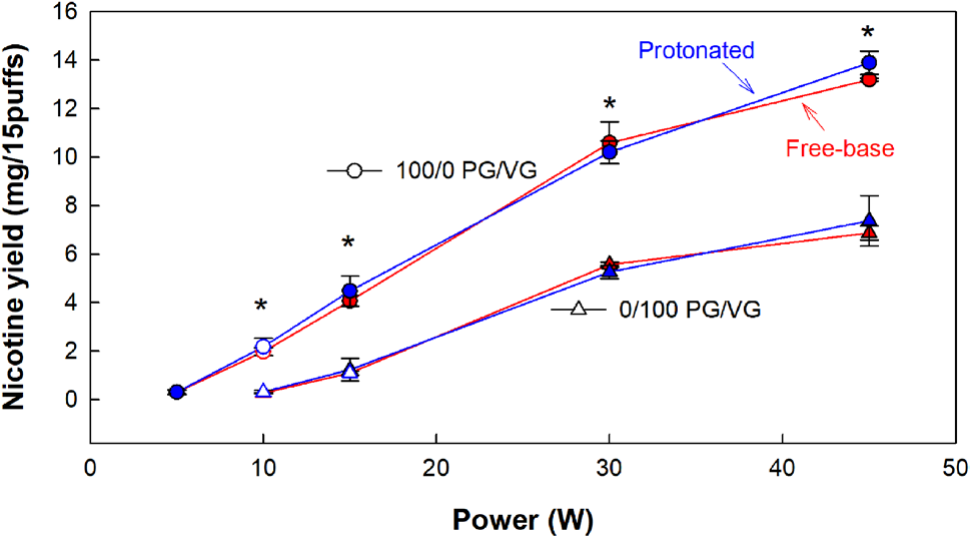
Average (± SD) nicotine yield (mg/15puffs) across conditions that differ by nicotine form, PG/VG ratio, and powers. Filled symbols indicate a significant difference from the 10 W condition, asterisks (*) indicate significant differences from the 0/100 PG/VG condition for each tested power.

Figure 1 shows the effect of nicotine form on nicotine yield at varying powers and PG/VG ratios. Nicotine yield was not significantly associated with nicotine form (*p* = 0.67), whereas yield was strongly associated with power and PG/VG ratio (*p* < 0.01). The regression model was found to explain 93% of the variance in nicotine yield (p < 0.01). We also found that nicotine yield can be predicted accurately from the product of the TPM and the liquid nicotine concentration (R^2^ = 0.92). A summary of the results is presented in Table 1.

**Table 1:** Mean (SD) of nicotine and TPM yields (N = 3) obtained using free-base and protonated nicotine at varying powers and PG/VG ratios.

PG/VG	Power (W)	Nicotine (mg/15puffs)		TPM (mg/15puffs)	
		Free-base	Protonated	Free-base	Protonated
100/0	5^a^	0.31 (0.10)	0.31 (0.07)	17.58 (9.35)	17.48 (3.49)
100/0	10	1.97 (0.16)	2.18 (0.34)	154.1 (10.81)	168.4 (44.48)
100/0	15	4.07 (0.23)	4.48 (0.61)	309.7 (12.3)	346.4 (48.56)
100/0	30	10.6 (0.87)	10.2 (0.47)	721.5 (42.75)	748.75 (62.82)
100/0	45	13.2 (0.06)	13.9 (0.48)	941.5 (16.04)	956.25 (36.4)
0/100	10	0.27 (0.03)	0.31 (0.06)	7.15 (1.78)	3.15 (1.3)
0/100	15	1.09 (0.08)	1.23 (0.47)	59.65 (14.64)	55.25 (44.51)
0/100	30	5.57 (0.1)	5.27 (0.27)	547.25 (25.7)	501 (56.51)
0/100	45	6.88 (0.29)	7.38 (1.04)	799.5 (8.11)	793.5 (33.85)

^a^While the lowest power used for the PG liquid was equal to 5 W, this power level was insufficient to generate a quantifiable amount of aerosol using the VG liquid. Thus for the VG condition 10 W was the minimum power used.

## Discussion

This study investigated the effects of protonated vs. free-base nicotine on nicotine yield, at varying powers and PG/VG ratios. We found that nicotine yield was not associated with nicotine form, but that yield increased with power and when the liquid vehicle was PG.

The null effect of nicotine form on yield has been reported previously for combustible cigarettes^14^, and is consistent with the notion that when heated, protonated nicotine undergoes dissociation of the acid/base pair during vaporization and then recombines upon condensation of the aerosol^3,10^. We have previously shown that the nicotine form in the ECIG aerosol corresponds to that of the liquid^11^. While form does not impact yield, it likely affects user sensory experience, such as the “throat hit” of the inhaled aerosol^3^.

The strong effect of liquid vehicle and power is consistent with previously presented theory of ECIG operation^8^, and with previous empirical studies^9,15^. In brief, the nicotine vaporization rate depends on the rate at which the liquid vehicle vaporizes from the ECIG heating coil^8^. The ECIG liquid vaporizes either by evaporation, when the temperature of the liquid is below its boiling temperature, or boiling, when the temperature of the liquid is equal to its boiling temperature. In the evaporation regime, the vaporization rate is governed by the volatility of the liquid. Because PG is more volatile (i.e., has a lower boiling point) than VG, the PG condition will produce a higher vaporization rate at a given temperature and power. In the boiling regime, the amount of liquid vaporized depends on how much of the thermal energy produced by the coil reaches the liquid versus being lost to the surroundings. The rate of thermal energy loss to the surroundings is, in turn, proportional to the temperature difference between the heating coil and the ambient surroundings. Because VG has a higher boiling temperature than PG, the temperature that the system reaches with VG is greater than with PG, and results in greater energy losses to surroundings, and less energy delivery to the liquid. Therefore, in both evaporation and boiling regimes (equivalent to low and high power for a given device), PG vaporizes at a greater rate than VG, carrying more nicotine per unit time^8^.

However, while PG-based liquids result in higher nicotine yield than VG liquids, the latter are commonly used in ECIG products, particularly in sub-Ohm devices and pod-systems, such as JUUL (30/70 PG/VG ratio^16^). Apart from nicotine, other factors that contribute to the ECIG desirability include the making possible the ability to exhale “big clouds” of aerosol^17^. This feature of ECIG operation is associated with the PG/VG ratio^15^. Compared to PG, VG produces larger particles that are capable of scattering more light, resulting in a more visible aerosol^15^. This factor, combined with greater throat irritation, likely contributes to the lower overall satisfaction associated with PG-based liquids and higher preference for VG-based liquids^18^.

In summary, we found that for a fixed puffing protocol, nicotine form is unlikely to influence yield for any current practical scenario (i.e., actual user power levels). However, while form does not affect yield, it may affect nicotine delivery to the blood. To date, research available on the effect of nicotine form on nicotine delivery shows contradictory outcomes^19,20^, potentially from the different methods used^21^. In addition, to the extent that nicotine form modifies sensory experience, it may also influence puffing and inhalation behavior and therefore exposure. For example, previous studies have shown that users modified their puffing behavior (i.e., lower puff duration and volume) when using PG instead of VG-based liquids, likely due to sensory experience^18^. We speculate that nicotine form may play a similar role; for example, users may decrease puffing intensity when using free-base nicotine, and thereby obtain less nicotine. Controlled clinical studies on the impact of form on puffing behavior and exposure could address these questions.

## Methods

### Aerosol generation

Aerosol was generated using the American University of Beirut Aerosol Lab Vaping Instrument (ALVIN), a custom-built digital puffing machine that can replicate in high resolution individual human puffing behavior. A Kanger Subox Mini-C tank ECIG was connected to ALVIN to generate the aerosol samples. The Subox tank was fitted with a coil head from the same manufacturer (SSOC nichrome 0.5 Ω) and powered using a DC power supply. Five power levels were used in the range 5–45 W. The powers were selected to cover a wide range within the device’s operating power output (1–50 W). For each sampling session, the aerosol exiting the mouth end of the ECIG was drawn through a Gelman type A/E 47 mm glass fiber filter pad where the particle phase of the aerosol was trapped. Total particulate matter (TPM) was determined by weighing the filter assembly before and after each session. Puff topography conditions were kept constant across all conditions at 4 s puff duration, 10 s interpuff interval, and 8LPM flow rate, approximating the average flow rate obtained in a clinical setting using the same device as reported by Hiler et al.^22^. Three new ECIG heater coils were used for each of the four liquid formulations (i.e., 12 coils in total were used for this study). Each coil was used to sample aerosol at all powers. A detailed list of conditions is provided in Table 1.

### ECIG liquid preparation

Analytical grade PG (≥ 99.5%, CAS 57-55-6), VG (99.0–101.0%, CAS 56-81-5), nicotine (≥ 99%, CAS number 54-11-5) and benzoic acid (≥ 99.5%, CAS 65-85-0) were procured from Sigma-Aldrich Corporation and used to prepare four different solutions with entirely free-base or protonated nicotine at 15 mg/g concentration, each using a 100/0 and 0/100 PG/VG ratios (Table 1). These ratios were selected to test the maximum range of potential liquid vehicle interaction with the effect of nicotine form on nicotine yield. The protonated nicotine solutions were prepared by adding standard solutions of benzoic acid to free-base nicotine as 1:1 mol ratio.

### Nicotine quantification

Nicotine in the aerosol and liquid was determined by GC–MS analysis of samples extracted in an ethyl-acetate solvent as previously described in Talih, et al.^23^. An extracted calibration curve with concentrations ranging from 1 to 20 ppm and spiked with the internal standard hexadecane was used to interpret the resulting chromatograms. Spiked filter assays of nicotine in PG and VG solutions showed recoveries of > 90%.

### Statistical analysis

A multiple linear regression analysis was used to estimate the association between nicotine yield with nicotine form, power, and PG/VG ratio. Statistical analyses were performed using SPSS version 25.0 (IBM, Armonk, NY, USA). Statistical significance was *p* < 0.05.
